# Artificial Intelligence with Robotics for Metabolic Rehabilitation and Enhanced Patient Recovery in Critical Care

**DOI:** 10.34133/research.1302

**Published:** 2026-06-26

**Authors:** Yisheng Chen, Guanghui Wu, Lili Yin, Ye Ding, Liming Zhu, Jing Cui, Hua Chen, Zhiwei Li, Shaocong Zhao, Haojun Shi, Jinjing Xia, Jian Guo, Lei Huang, Lihua Dai

**Affiliations:** ^1^ Fujian Key Laboratory of Toxicant and Drug Toxicology, Medical College, Ningde Normal University, Ningde, China; Ningde Normal University, Ningde, China; Department of Vascular and Interventional Radiology, Ningde Municipal Hospital of Ningde Normal University, Ningde, China; Fujian Key Laboratory of Medical Bioinformatics, Fujian Medical University, Fuzhou, China.; ^2^ Department of Emergency, Shidong Hospital of Yangpu District, Shanghai, China.; ^3^ Ningde Municipal Hospital of Ningde Normal University; Ningde Clinical Medical College of Fujian Medical University, Fuzhou, China.; ^4^ Emergency and Intensive Care Unit, Shidong Hospital Affiliated to University of Shanghai for Science and Technology, Shanghai, China.; ^5^ Yrobot Inc., Suzhou, China.; ^6^ School of Life Sciences, Fudan University, Shanghai, China.; ^7^ Department of Critical Care Medicine, Shidong Hospital of Yangpu District, Shanghai, China.; ^8^ Faculty of Science, University of Malaya, Kuala Lumpur, Malaysia.; ^9^ Clinical Laboratory Center, The People’s Hospital of Xinjiang Uygur Autonomous Region, Xinjiang Uygur Autonomous Region, China.; ^10^ Xiamen University of Technology, Xiamen, China.; ^11^ Faculty of Chinese Medicine and State Key Laboratory of Quality Research in Chinese Medicines, Macau University of Science and Technology, Macau, Macau SAR, China.; ^12^ Department of Respiratory and Critical Care Medicine, Shanghai Chest Hospital, Shanghai Jiao Tong University, Shanghai, China.; ^13^ Department of Pulmonary Function Test, Shanghai Pulmonary Hospital Affiliated to Tongji University, Shanghai, China.; ^14^ Department of Molecular Cell and Cancer Biology, University of Massachusetts Chan Medical School, Worcester, MA, USA.

## Abstract

This narrative review summarizes recent advances in the integration of artificial intelligence (AI)-driven rehabilitation robotics with metabolic regulation in critically ill pulmonary patients. AI-enabled robotic systems, combining multimodal physiological sensing with adaptive machine learning, allow continuous monitoring of cardiopulmonary and metabolic parameters and support individualized, dynamic interventions. Unlike conventional rehabilitation based on fixed protocols, these systems establish closed-loop feedback between metabolic signals and motor output, enabling sustained low-intensity muscle activation while optimizing oxygen utilization, glucose metabolism, and mitochondrial function. Such regulation may interrupt the pathological interplay among inflammation, metabolic imbalance, and muscle atrophy, thereby promoting respiratory and systemic recovery. Recent developments in metabolic monitoring, biofeedback control, and multi-omics integration have further extended these platforms toward comprehensive metabolic management. By integrating biomechanical support with computational and biochemical intelligence, this approach reframes rehabilitation as an active process of metabolic reprogramming. However, current evidence remains heterogeneous, and well-designed clinical studies are needed to validate the reproducibility and clinical efficacy of these strategies.

## Introduction

The clinical management of severe pulmonary diseases such as acute respiratory distress syndrome (ARDS) and refractory pneumonia has reached a point where acute survival is no longer the sole therapeutic objective. While advancements in life support have improved short-term survival rates, they have also unveiled a systemic challenge known as post-intensive care syndrome, in which survivors experience profound and often irreversible declines in physical, respiratory, and cognitive function [1]. A fundamental driver of this functional decline is systemic bioenergetic failure characterized by a persistent hypercatabolic state alongside impaired mitochondrial function and increased oxidative stress. These metabolic disturbances compromise respiratory mechanics while reducing muscle endurance and creating a physiological environment where conventional rehabilitation efforts are frequently insufficient. The consequences are further compounded in patients subjected to prolonged mechanical ventilation or extended bed rest, which results in accelerated skeletal muscle wasting and profound insulin resistance. These clinical observations indicate that effective pulmonary rehabilitation must extend beyond simple physical mobilization to actively address metabolic reprogramming as a central therapeutic target.

**Fig. 1.**
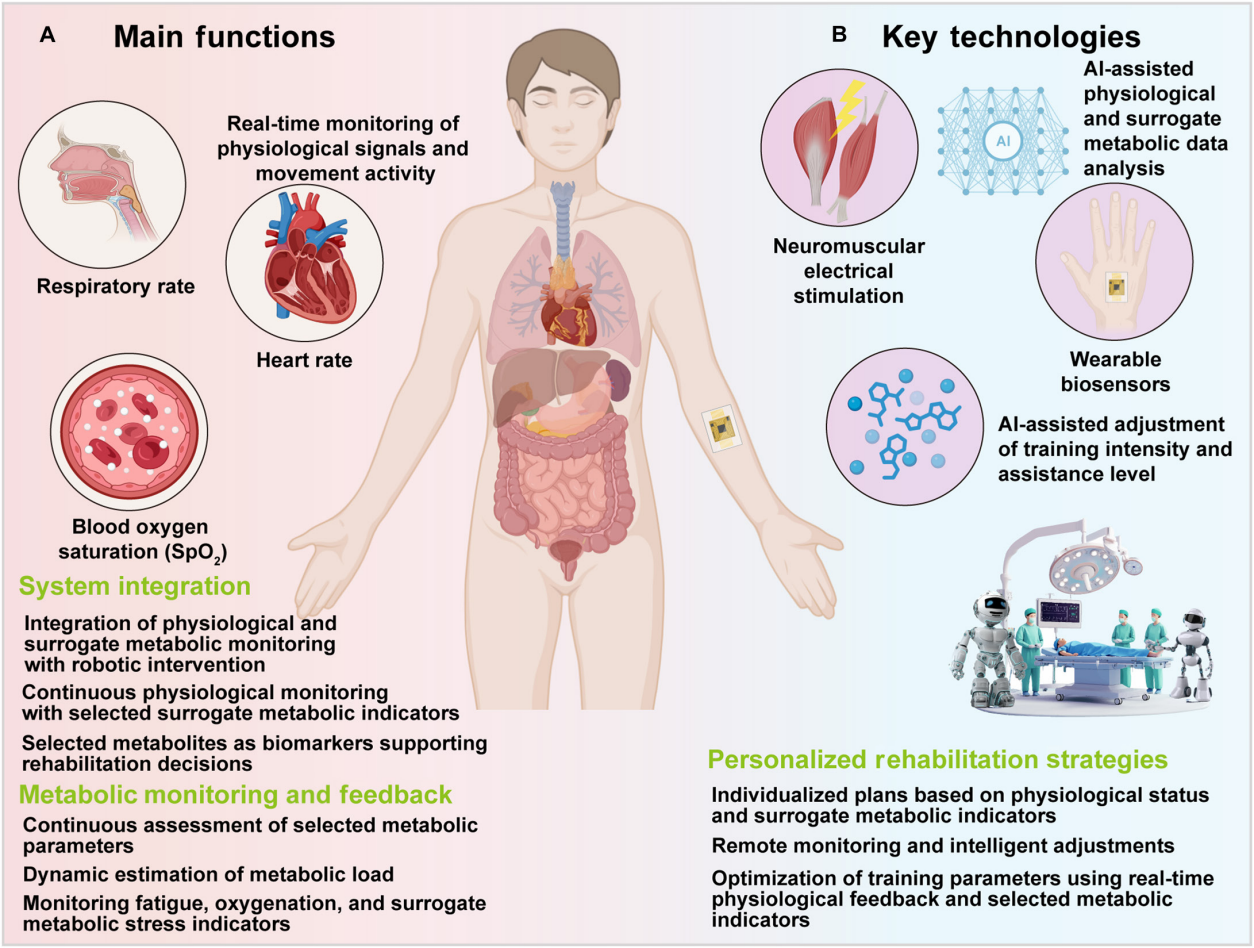
Applications of rehabilitation robots for metabolic regulation in critically ill patients with pulmonary diseases. (A) Main functions: A wearable system tracks vital signs such as respiratory rate, heart rate, and blood oxygen levels. It monitors metabolic parameters in real time to regulate oxidative stress and support tissue repair during rehabilitation. This system uses metabolites as biomarkers to guide and personalize treatment. (B) Key technologies: Key technologies include AI-driven analysis for personalized care, wearable biosensors for continuous monitoring, and neuromuscular electrical stimulation. When coupled with remote monitoring and intelligent adjustment, robotic interventions, including those applied in perioperative and early postoperative settings, can optimize rehabilitation based on real-time data.

Conventional pulmonary rehabilitation strategies, including manual physiotherapy and guided breathing exercises, face substantial limitations when applied to critically ill populations. The dynamic and fragile physiological state of these patients creates a substantial intervention–quantification gap, in which the absence of high-fidelity feedback prevents the precise titration of exercise intensity [2]. In clinical practice, undertreatment may fail to stimulate necessary muscle activity while excessive intervention risks exercise-induced exhaustion or hemodynamic instability. These challenges are particularly pronounced during mechanical ventilation when respiratory muscles may be unloaded or underactivated and the risk of intensive care unit (ICU)-acquired weakness is high. Intelligent rehabilitation robotics offers a potential solution to this challenge by integrating biomechanical assistance with real-time sensing and feedback mechanisms (Fig. 1) [3]. Robotic platforms can maintain muscular integrity and enhance ventilation efficiency through controlled neuromuscular stimulation [4]. They enable the precise modulation of exercise intensity and frequency, which facilitates early and safe intervention even in patients with severely limited mobility. The evolution of these platforms reflects a shift toward a data-driven paradigm in pulmonary rehabilitation where every intervention can be quantified and adjusted in response to patient-specific physiological signals [5]. Through integrated sensor systems, rehabilitation robots provide real-time monitoring of heart rate, respiratory parameters, and muscle activation, allowing for a level of precision that is difficult to achieve with conventional manual therapy [6]. Such platforms can deliver both passive and active training modalities, supporting individualized rehabilitation programs and demonstrating potential in mitigating muscle atrophy and improving respiratory function [7,8]. Despite these advantages, most existing robotic platforms remain metabolically blind because they primarily follow preprogrammed kinematic trajectories and lack the ability to interpret real-time biochemical or energetic signals from the patient [9]. As a result, even advanced robotic systems cannot autonomously adjust training loads in response to changing oxidative stress levels or increased protein catabolism. This limitation restricts their effectiveness in the most complex clinical scenarios where metabolic dysregulation is the dominant barrier to recovery.

Failure of early rehabilitation in severe pulmonary disease is closely linked to the pathological interplay known as the lung–muscle axis [10]. Systemic inflammation and chronic hypoxia converge to impair mitochondrial function, which favors protein catabolism over protein synthesis. This metabolic derangement manifests as progressive muscle wasting and reduced energy availability, thereby limiting the effectiveness of conventional mechanically driven rehabilitation interventions. Without synchronization between physical load and cellular bioenergetics, traditional rehabilitation may inadvertently exacerbate the inflammatory cycle and further impede recovery. Addressing metabolic dysregulation is therefore not a supplementary goal but a decisive bottleneck that determines whether patients can transition from mechanical dependence to functional independence. Interventions that support mitochondrial function and restore energy balance are essential for enabling effective rehabilitation. Understanding the mechanisms underlying the lung–muscle axis is critical for designing strategies that coordinate mechanical training with metabolic restoration.

Translating these pathophysiological insights into clinically actionable rehabilitation strategies, however, remains challenging, highlighting several key limitations in current practice. First, bedside sensing technologies capable of capturing real-time metabolic information are still limited, and computational frameworks for processing multimodal physiological time-series data remain insufficiently developed. Moreover, the absence of a unified framework for integrating metabolic signals with robotic control continues to limit the realization of truly closed-loop rehabilitation systems. In an optimal scenario, rehabilitation interventions would be dynamically modulated based on continuously acquired physiological and surrogate metabolic signals. Such an approach would enable patients to remain within an individualized metabolic safety range while balancing oxygen delivery and energy utilization and limiting fatigue accumulation under critical care conditions [11]. Collectively, these challenges reveal a critical gap between current technological capabilities and the clinical demands of metabolically informed rehabilitation in critically ill pulmonary patients. This gap underscores the need for a structured, disease-oriented framework to guide future development. To address this need, the present review proposes a conceptual framework for metabolically synchronized rehabilitation, with a particular focus on critically ill pulmonary populations, among whom metabolic dysregulation and the lung–muscle axis represent central determinants of recovery. This framework is grounded in a structured synthesis of existing evidence identified through a comprehensive literature search of major electronic databases, including PubMed, Embase, Web of Science, Scopus, and IEEE Xplore, covering the period from January 2000 to December 2025. The search strategy combined Medical Subject Headings and free-text terms related to rehabilitation robotics, pulmonary and critical care rehabilitation, metabolic regulation, and artificial intelligence (AI), using Boolean operators to optimize retrieval. Eligible studies included original research, clinical investigations, and relevant reviews in these domains, while studies not related to pulmonary disease or metabolic mechanisms were excluded. Additional relevant literature was identified through manual screening of reference lists. Given the interdisciplinary and rapidly evolving nature of this field, the evidence was synthesized narratively to facilitate cross-domain integration. Within this framework, particular attention is given to the integration of multimodal physiological sensing with emerging metabolic assessment approaches, including metabolomics and exhaled breath analysis, as complementary layers for characterizing patient status. Rather than serving as direct real-time control inputs, these modalities provide context for understanding metabolic adaptability and recovery trajectories. Their integration with adaptive robotic control highlights a pathway toward more responsive and physiologically aligned rehabilitation strategies, supporting the transition from experience-based protocols toward precision, data-informed clinical management in critically ill pulmonary patients.

## Application of Rehabilitation Robots in Pulmonary Disease Rehabilitation

### Evolution and background of rehabilitation robot technology

**Fig. 2.**
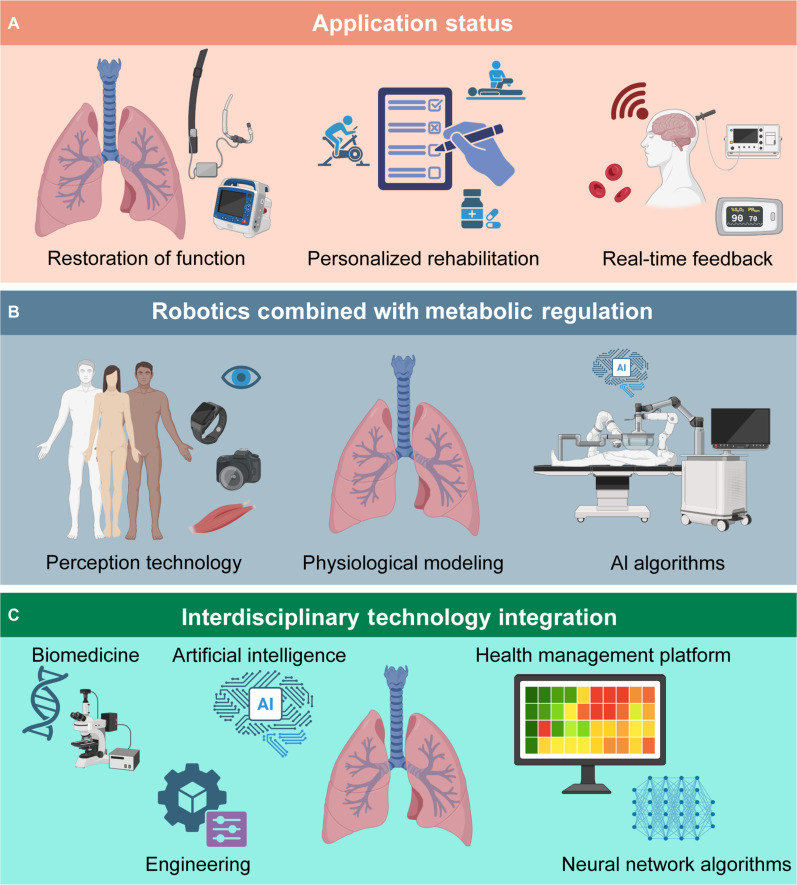
Applications of rehabilitation robots for pulmonary diseases. (A) Application status: Rehabilitation robots help restore function and provide personalized care in the treatment of pulmonary diseases. They offer real-time feedback and adjust treatment as needed to optimize recovery and respiratory function. (B) Robotics combined with metabolic regulation: This module combines robotics with metabolic regulation. Perception technology monitors body responses, while AI analyzes real-time data to guide rehabilitation. Physiological modeling helps optimize rehabilitation strategies based on metabolic responses for better recovery. (C) Interdisciplinary technology integration: This section highlights the integration of AI, biomedicine, and engineering in pulmonary rehabilitation. Neural network algorithms and health management knowledge graphs improve decision-making and personalized care, enhancing treatment and long-term health management.

Since its inception at the end of the 20th century, rehabilitation robot technology has emerged as a cornerstone of clinical and translational research [12]. Initial applications primarily targeted the restoration of motor function in patients with neurological disorders by providing mechanical assistance to facilitate repetitive training and improve limb mobility. However, the rapid advancement of AI, sensor technologies, and human–machine interaction systems has catalyzed a fundamental shift in this field. These devices have evolved from monofunctional mechanical actuators into autonomous intelligent systems capable of sophisticated perception, data analysis, and clinical decision supportand are now widely applied in the management of chronic and critically ill populations [13]. In the context of pulmonary diseases, especially in critically ill patients with pulmonary diseases, conventional therapies often struggle to reconcile systemic physiological adaptability with the requirement for continuous intervention. These patients typically present with severe respiratory dysfunction, systemic metabolic abnormalities, and disuse muscle atrophy, all of which necessitate a rehabilitation paradigm that is personalized, precise, and dynamically adjustable. In this setting, rehabilitation robots offer unique advantages by automating respiratory training and enhancing pulmonary function while integrating multidimensional physiological monitoring. This synergy allows for the real-time assessment of metabolic status and intervention adjustment based on real-time metabolic information, thereby providing a novel trajectory for systematic functional recovery (Fig. 2).

Furthermore, rehabilitation robots can improve pulmonary ventilatory function and respiratory muscle endurance by titrating training rhythm and intensity, thereby reducing the incidence of secondary pulmonary complications [14]. Leveraging integrated multimodal sensing systems, these robots continuously acquire critical parameters such as blood oxygen saturation, respiratory rate, and heart rate to formulate individualized programs aligned with a patient’s physiological tolerance. During early intervention phases, robotic systems safely initiate functional activity in bedridden individuals to reduce muscle catabolism and mitigate tissue depletion in high metabolic states [15]. Compared to traditional methods, these platforms improve standardization and provide quantitative feedback that supports evidence-based strategies [12]. Their intelligent adaptability enables interdisciplinary integration, moving rehabilitation from experience-driven models toward data-driven paradigms that offer robust support for metabolic regulation and functional recovery in critically ill pulmonary patients.

### Current status of rehabilitation robot applications in pulmonary diseases

In recent years, rehabilitation robots have emerged as an important adjunct in the management of pulmonary diseases, particularly in patients affected by prolonged immobilization or severe infections [16]. These systems enable individualized rehabilitation through precisely controlled early mobilization and respiratory training, with the aim of improving respiratory mechanics, enhancing muscle metabolic capacity, and increasing exercise tolerance. This role is particularly relevant in critically ill patients, in whom systemic inflammation, hypoxia, and metabolic dysregulation contribute to rapid atrophy of both skeletal and respiratory muscles [17]. Within this pathophysiological context, structured robotic interventions may help mitigate muscle deterioration by promoting controlled muscle activation and supporting mitochondrial function, thereby improving energy metabolism and oxygen utilization efficiency. From a technological perspective, the integration of multimodal sensor systems enables real-time monitoring of physiological parameters, providing an objective basis for dynamically adjusting training intensity while maintaining safety and specificity [18]. Clinically, studies in pulmonary and critically ill populations have reported that robotic-assisted rehabilitation is associated with improvements in ventilatory parameters and pulmonary compliance. In addition, findings from broader rehabilitation and exercise physiology research suggest potential effects on metabolic biomarkers, including markers of glucose and lipid metabolism [19]. When combined with metabolic monitoring tools, robotic platforms may further enable the assessment of metabolic indicators such as lactate and selected intermediary metabolites, offering additional insight into patient-specific metabolic responses. Despite these advances, several important limitations remain. Direct clinical evidence supporting metabolically integrated robotic rehabilitation in critically ill pulmonary patients is still limited, and the field remains at an early stage of development [20]. Much of the available evidence is derived from general rehabilitation or exercise physiology populations, which constrains its direct applicability to critically ill patients. Current robotic systems also tend to prioritize mechanical output, with only limited incorporation of metabolic feedback into control strategies [21].

**Fig. 3.**
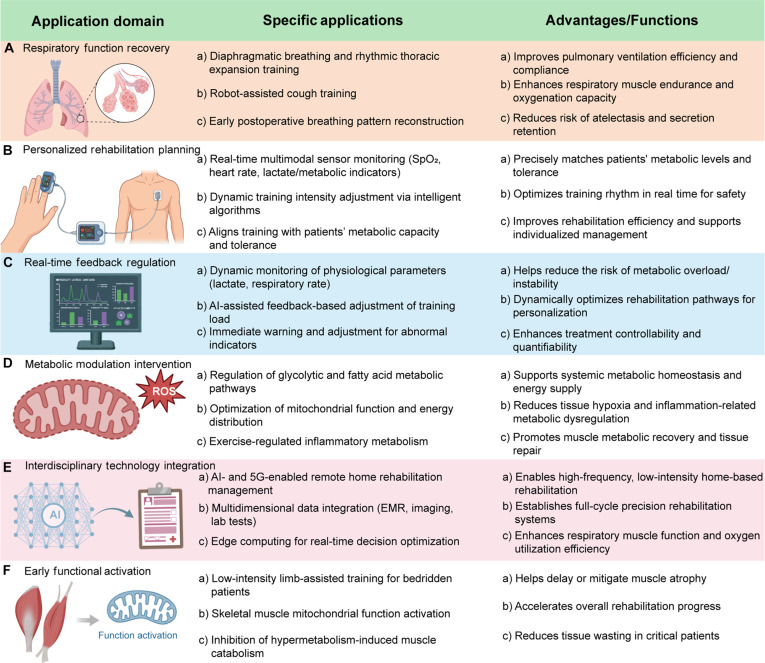
Application of rehabilitation robots in pulmonary disease recovery. (A) Respiratory function recovery: This module improves respiratory function through breathing exercises and robot-assisted cough training. It boosts pulmonary ventilation, muscle endurance, and oxygenation, while reducing the risk of atelectasis and secretion retention. (B) Personalized rehabilitation planning: This module uses real-time sensor data (SpO_2_, heart rate, and metabolites) to create tailored rehabilitation plans. Algorithms adjust training intensity based on metabolic tolerance, ensuring safe and efficient rehabilitation. (C) Real-time feedback regulation: This module tracks parameters like lactate and respiratory rate. AI-based systems adjust training loads when needed, helping reduce the risk of metabolic issues and personalizing rehabilitation. (D) Metabolic modulation intervention: This module activates metabolic pathways to optimize mitochondrial function and regulate inflammation. These actions improve energy balance, reduce hypoxia, and support muscle recovery. (E) Interdisciplinary technology integration: This module uses AI and 5G for remote rehabilitation. It integrates data (EMR, imaging, and lab tests) and edge computing to optimize real-time decisions and enhance respiratory muscle function. (F) Early functional activation: This module focuses on low-intensity training for bedridden patients. It activates muscle mitochondria, helps reduce muscle loss, and supports recovery.

Impaired respiratory function represents a central complication in critically ill pulmonary patients and often disrupts systemic metabolic balance [22]. Rehabilitation robots provide technical support by coupling targeted respiratory muscle training with individualized planning during critical and postoperative phases. By integrating sensor-based feedback and visual guidance with controlled training loads, these robots facilitate respiratory muscle strengthening through diaphragmatic breathing and rhythmic thoracic expansion [23]. These systems enable early functional activity during the post-acute phase to aid in secretion clearance and reduce the risk of atelectasis. For patients with limited motivation due to pain, robotic assistance improves training adherence and minimizes ineffective muscle strain [24]. Robotic platforms facilitate systematic respiratory muscle training in combination with lower limb endurance exercises, thereby yielding measurable improvements in respiratory performance within abbreviated timeframes. The integration of electromyography feedback and load modulation ensures that interventions are both individualized and quantifiable [25]. Personalized plans rely on real-time physiological and surrogate metabolic data, such as blood oxygen saturation, heart rate, and lactate levels, to dynamically adjust training intensity (Fig. 3). Intelligent algorithms utilize these inputs to tailor metabolic load-appropriate programs in which patients with unstable metabolism begin with low-intensity intermittent protocols before progressing to higher loads [26]. Machine learning further refines intervention logic over time by analyzing longitudinal trends to optimize program design [27]. This approach enhances safety and helps reduce the risk of metabolic instability, integrating respiratory recovery with metabolic regulation for a holistic strategy.

Real-time feedback mechanisms are essential for individualized and adaptive rehabilitation in pulmonary critical care. Rehabilitation robots function as advanced systems that combine intelligent perception and control technologies to demonstrate substantial clinical utility [28]. Through built-in sensors, these robots continuously monitor physiological parameters such as respiratory rate and blood oxygen saturation [29]. These data inform instantaneous adjustments to intervention strategies to ensure optimal therapeutic conditions (Fig. 3). Technological advancements allow robots to respond to a patient’s dynamic status with high precision by integrating imaging and intelligent algorithms to support clinical decision-making [30,31]. The application of robotic technologies in bronchoscopy and pulmonary nodule biopsy illustrates this integration, in which visualization and navigation functions facilitate stable access to complex airways [32,33]. High controllability in minimally invasive procedures may reduce complications and improve the quality of tissue acquisition [34]. By incorporating real-time feedback, these systems provide quantifiable metrics for postoperative assessment. With improvements in intraoperative image processing and data analysis, robotic systems assist physicians in rapid decision-making and continuous optimization of postoperative plans [31,35,36]. Machine learning and AI further enhance the identification of tissue structures to support dynamic adjustments [37]. This human–machine physiological closed-loop system shifts rehabilitation from experience-driven to data-driven models to support better metabolic regulation and aid pulmonary recovery. Future research should prioritize processing efficiency and multimodal data integration to maintain high-precision monitoring in complex environments [38,39].

### Integration of robotics and metabolic regulation

Rehabilitation robots are increasingly transitioning from experience-based interventions toward more precise and intelligent regulation in the management of pulmonary diseases (Fig. 3). Within this evolving paradigm, metabolic regulation has emerged as a central component of pulmonary rehabilitation, contributing to both systemic recovery and the restoration of lung function. Firstly, the clinical relevance of metabolic regulation is particularly evident in critically ill patients, in whom prolonged immobility and mechanical ventilation are frequently associated with profound disruptions in energy metabolism and severe muscle atrophy [40]. Exercise-based interventions have been shown to enhance energy utilization and promote tissue repair through mechanisms such as glycolytic activation and improved mitochondrial function. However, much of the supporting mechanistic evidence originates from non-critically ill populations, and its applicability to critically ill pulmonary patients remains to be further validated [41,42]. Secondly, from an implementation perspective, rehabilitation robots provide a platform for translating these metabolic principles into controlled clinical interventions. Systems equipped with integrated sensors enable continuous monitoring of physiological and surrogate metabolic indicators, including lactate levels and carbon dioxide elimination, thereby providing dynamic data to guide exercise adjustment. Through this capability, robotic platforms can dynamically adapt operational parameters to patient-specific conditions, forming a monitoring–feedback–adjustment loop that supports individualized rehabilitation. In addition, multimodal bedside data, including vital signs, ventilatory parameters, gas exchange indices, and surrogate metabolic markers such as lactate trends and muscle oxygenation, can be integrated to align robotic assistance with the patient’s evolving metabolic state [43]. Within this integrated framework, structured robotic-assisted activity may influence multiple metabolic pathways and has been associated with enhanced fat oxidation and reduced inflammation-related metabolic disturbances. Finally, advances in machine learning further extend this framework by enabling the identification of metabolic profiles and the prediction of patient-specific responses to training. These approaches support the refinement of rehabilitation strategies and contribute to the development of more adaptive and personalized interventions. Collectively, these developments in sensing, physiological modeling, and computational intelligence reinforce the role of robotic systems in enabling personalized, metabolically informed pulmonary rehabilitation.

### Interdisciplinary technological integration

The rapid advancement of biomedical engineering and AI has substantially expanded the role of rehabilitation robots in metabolic regulation. Through the integration of mechanical systems and physiological monitoring, these robots have evolved from simple motion-assistive devices into intelligent, multidimensional interactive platforms (Fig. 3). AI, particularly deep learning and neural network techniques, enables the effective integration of physiological data and supports the dynamic adjustment of rehabilitation protocols [44,45]. Based on this foundation, recent studies have explored advanced computational approaches, including deep learning-based biomechanical modeling, reinforcement learning-driven control strategies, and transformer-based frameworks. These methods further improve trajectory control and adaptive human–robot interaction in rehabilitation systems [46–50]. These systems continuously monitor fluctuations in patient status and provide corresponding adjustment recommendations, thereby supporting improvements in the precision and safety of rehabilitation processes. In addition, AI integrates electronic medical records and medical imaging data to enable more comprehensive and continuous health management. Through interdisciplinary collaboration, the combination of biological signal recognition and behavioral feedback further enhances clinical adaptability, allowing rehabilitation robots to dynamically adjust interventions according to individual differences [51,52].

Emerging technologies such as 5G and edge computing support remote rehabilitation management by enabling cloud- and edge-based analysis for home rehabilitation programs [53]. High-frequency and low-intensity personalized load adjustments improve oxygen utilization and promote respiratory muscle reconditioning to support pulmonary function recovery from a metabolic perspective. In summary, rehabilitation robots have evolved into comprehensive platforms that integrate metabolic regulation and intelligent perception. Their underlying logic reflects both engineering advancements and deep biomedical understanding, marking a major shift toward intelligent and continuous pulmonary rehabilitation.

## The Mechanistic Role of Metabolites in Robotic-Assisted Pulmonary Recovery

### Bioenergetic reprogramming and mitochondrial resilience

**Fig. 4.**
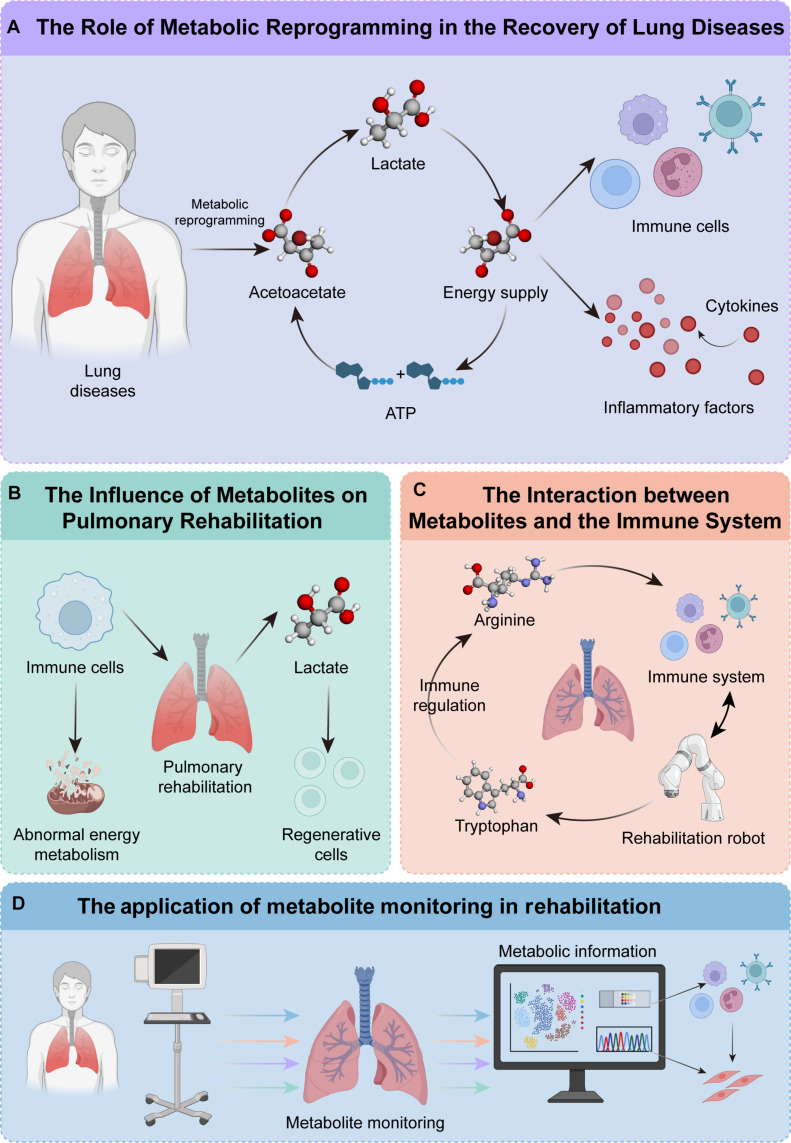
The role of metabolites in the rehabilitation of lung diseases. (A) Metabolic reprogramming in lung disease recovery: This module explains how metabolic reprogramming aids lung disease recovery. Metabolites like lactate and acetoacetate provide energy to immune cells and tissues. These changes support lung regeneration, inflammation control, and cellular repair. (B) Metabolites in pulmonary rehabilitation: This section shows how metabolites help lung repair during rehabilitation. They support oxygen utilization, balance metabolic pathways, and modulate immune responses, improving rehabilitation. (C) Metabolites and immune system interaction: This module explores how metabolites like lactic acid and arginine affect immune cells. These metabolites balance inflammation and repair. Rehabilitation robots adjust therapy based on metabolite-driven immune responses, supporting recovery. (D) Metabolite monitoring in rehabilitation: This section highlights the value of metabolite monitoring. Continuous metabolic data track recovery and guide treatment. This helps support personalized care and may improve outcomes for lung disease patients.

Metabolic reprogramming represents a fundamental physiological basis for recovery in critically ill patients, in whom the lung–muscle axis functions as a key pathway for systemic restoration [54]. At the mechanistic level, this process is thought to involve alterations in energy metabolism and associated signaling pathways. However, current insights are primarily derived from preclinical and basic research, and their direct clinical applicability in critically ill patients with pulmonary dysfunction remains to be fully established. The restoration of injured lung tissue is a highly energy-dependent process that requires precise allocation of adenosine triphosphate to support cellular repair and immune regulation (Fig. 4) [55]. In this context, robotic-assisted mobilization applies controlled mechanical loading that may activate mTORC1-related signaling pathways in both skeletal and respiratory muscles. Evidence from preclinical and mechanistic studies suggests that such activation may promote a metabolic shift from proteolysis-dominant catabolism toward protein synthesis and anabolic processes [56]. This metabolic transition may further alleviate the bioenergetic deficits commonly observed in ICU-acquired weakness and may be associated with enhanced oxidative phosphorylation and reduced mitochondrial reactive oxygen species production [57]. These changes improve overall energy efficiency while maintaining therapeutic loading within a safe physiological range [58]. In addition, robotic rehabilitation systems provide a standardized framework for monitoring metabolic activity. Through real-time physiological signal feedback, they enable continuous adjustment of intervention intensity, helping maintain both safety and stability throughout the rehabilitation process [59].

### Lactate as a metabolic fulcrum and signaling mediator

Lactate functions not only as a metabolic substrate but also as an important signaling mediator, rather than merely being a by-product of anaerobic metabolism [60]. In severe respiratory failure, lactate accumulation reflects the combined effects of tissue hypoxia and increased glycolytic flux [61]. Under such conditions, robotic-assisted rehabilitation may provide consistent and quantifiable exercise stimuli that help create conditions conducive to the lactate shuttle mechanism, thereby potentially supporting the redistribution of lactate between glycolytic and oxidative tissues. This redistribution enables lactate to be oxidized to pyruvate for mitochondrial oxidation, contributing to improved systemic energy efficiency. Beyond its metabolic role, lactate also participates in cellular signaling. Experimental evidence suggests that lactate can modulate the inflammatory microenvironment through pathways involving GPR81, which may promote macrophage polarization toward an anti-inflammatory phenotype [62]. This coordinated metabolic and signaling effect may support the resolution of inflammation, limit fibroproliferative responses, and promote the repair of the alveolar–capillary barrier, thereby facilitating recovery in pulmonary injury.

### Systemic immune-metabolic synergy and the lung–muscle axis

**Fig. 5.**
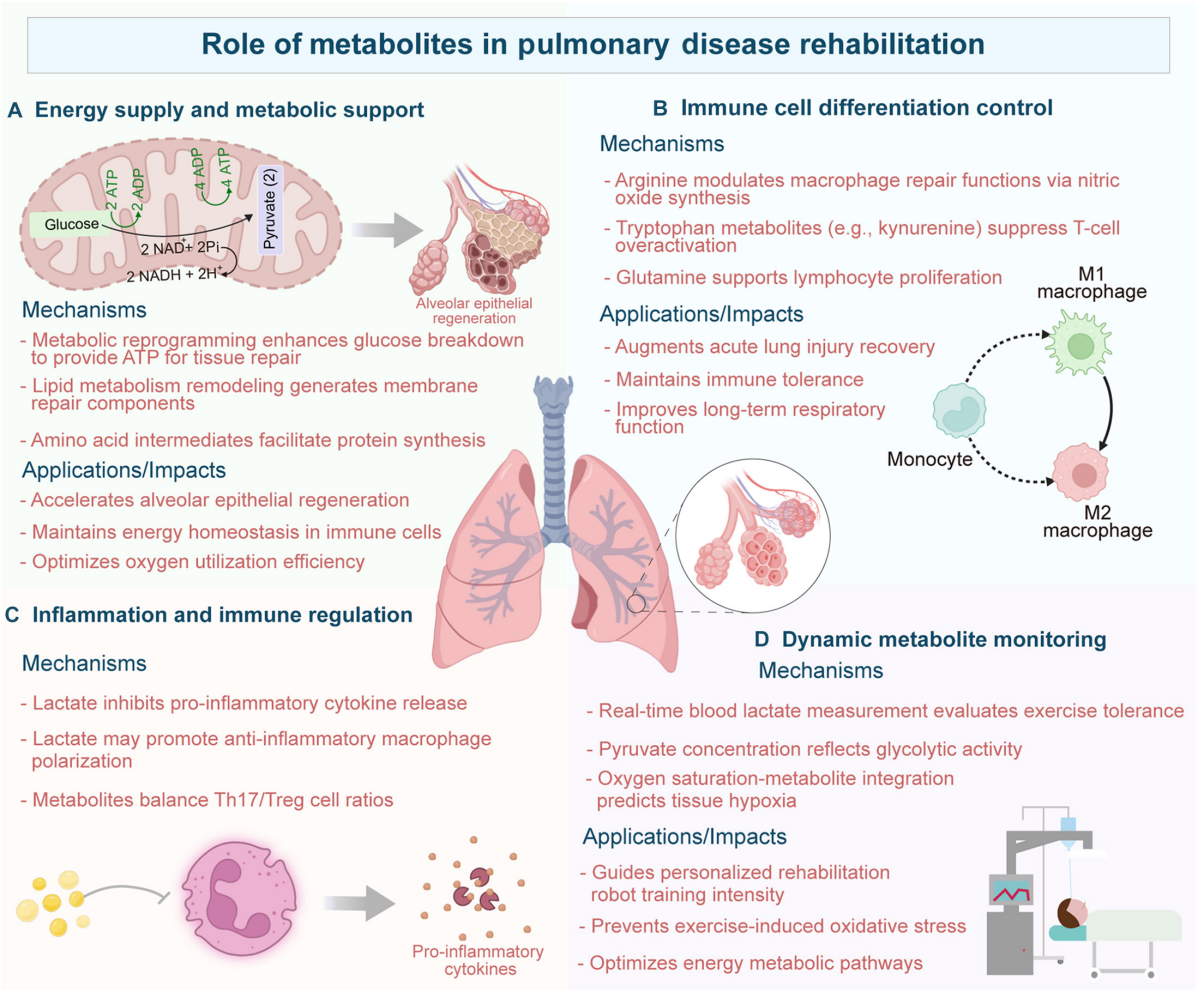
Role of metabolites in pulmonary disease rehabilitation. (A) Energy supply and metabolic support: This module explains how metabolism supports energy during pulmonary repair. Metabolic reprogramming enhances glucose breakdown, providing ATP for tissue repair. Lipid metabolism aids membrane repair. Amino acids help protein synthesis. These processes support lung regeneration, immune cell energy, and oxygen use, aiding lung function recovery. (B) Immune cell differentiation control: This section discusses how metabolites regulate immune cell differentiation. Arginine supports macrophage repair, tryptophan metabolites suppress T-cell activation, and glutamine aids lymphocyte growth. These processes help promote recovery from lung injury and improve long-term lung function. (C) Inflammation and immune regulation: This module explores how metabolites regulate inflammation and immune responses. Lactate may help attenuate inflammatory responses, while acetoacetate encourages anti-inflammatory macrophage activity. Metabolites balance Th17/Treg cells, promoting immune tolerance and lung recovery. (D) Dynamic metabolite monitoring: This module highlights dynamic metabolite monitoring. Real-time lactate measurement evaluates exercise tolerance, pyruvate shows glycolytic activity, and oxygen-metabolite integration helps predict hypoxia. This helps guide personalized rehabilitation, optimize training, and help prevent oxidative stress, enhancing recovery.

Effective pulmonary rehabilitation relies on the coordinated interaction between muscular activity and immune regulation [63]. Skeletal muscle acts as an endocrine organ during physical activity by secreting myokines, including interleukin-6 and irisin, which may contribute to the regulation of pulmonary metabolic homeostasis [64]. These myokines facilitate systemic metabolic responses by influencing the availability and metabolism of key amino acids such as arginine and tryptophan, both of which play essential roles in immune modulation [65]. Arginine supports macrophage-mediated tissue repair through nitric oxide synthesis, thereby counteracting epithelial dysfunction commonly observed in pulmonary diseases [66]. In parallel, tryptophan-derived metabolites contribute to immune tolerance by regulating immunometabolic pathways that balance inflammatory and reparative processes [67]. In addition, clinical studies have shown that early mobilization and structured physical activity are associated with the restoration of circulating amino acids such as glutamine and glycine, which serve as precursors for nucleotide synthesis and support cellular repair processes [68]. Together, these findings highlight the role of exercise-induced metabolic–immune coupling as a key mechanism underlying pulmonary recovery (Fig. 5). Within this mechanistic framework, rehabilitation robots provide a means of delivering structured and reproducible physical stimuli. By standardizing exercise intensity and timing, robotic-assisted interventions may help stabilize immune–metabolic networks, thereby supporting systemic metabolic adaptation, with potential effects on immune response modulation in both clinical and experimental settings [69].

### Advanced metabolite monitoring and closed-loop algorithmic control

Metabolomic analysis of exhaled breath condensate has been explored as a noninvasive approach for assessing metabolic alterations in patients with chronic obstructive pulmonary disease (COPD) [70]. In parallel, metabolic disturbances observed in chronic lung diseases have stimulated the development of bedside biosensing technologies capable of tracking metabolites such as lactate and glucose in a time-resolved manner [71]. These sensing modalities provide an expanding foundation for capturing dynamic metabolic and physiological states in clinical settings. From a rehabilitation engineering perspective, such metabolic and physiological measurements are more appropriately regarded as sources of supplementary contextual information rather than direct control inputs. Their primary value lies in supporting the interpretation of patient state and guiding individualized intervention strategies. Within this context, rehabilitation robots represent a potential platform for integrating these multimodal signals into clinical practice. When combined with robotic systems, metabolic and physiological data can be temporally aligned with movement patterns to distinguish active patient engagement from predominantly passive assistance, thereby enabling more informed adjustment of training parameters [72]. This integration provides the basis for developing monitoring–feedback–adaptation frameworks that support individualized rehabilitation.

Building on this concept, closed-loop regulation has been proposed as a potential strategy for incorporating physiological and metabolic information into rehabilitation control. Continuous monitoring of physiological signals may facilitate adaptive adjustment of robotic assistance, with the aim of improving safety and personalization. However, such approaches remain largely conceptual and have not yet been established as routine clinical practice, particularly in intensive care settings where implementation is constrained by technical and clinical factors. The conceptual information flow is schematically illustrated. In parallel, increasing attention has been directed toward computational methods for analyzing continuous physiological and metabolic data. These approaches aim to extract temporal features from time-series signals to support state assessment and optimization of rehabilitation strategies. Models such as long short-term memory networks have been applied in exercise science to characterize gradual metabolic changes associated with fatigue and may provide a basis for future rehabilitation monitoring [73]. Despite these advances, the integration of sensing technologies, robotic control, and computational modeling remains at an early stage. Most approaches represent emerging directions toward physiology-aware rehabilitation rather than standardized or clinically deployed solutions in intensive care environments.

## Integration of Multiomics Information into Rehabilitation Robotics for Metabolic Adaptation in Severe Pulmonary Disorders

Pulmonary diseases, including lung cancer, COPD, and ARDS, are recognized as major global health challenges. These conditions are frequently associated with systemic inflammatory responses, impaired tissue oxygenation, and marked metabolic disturbances, which collectively disrupt physiological homeostasis and hinder recovery capacity in critically ill patients [74]. As the concept of precision medicine continues to evolve, conventional symptom-based rehabilitation strategies are increasingly inadequate for addressing the multidimensional needs of these patients. Rehabilitation robots, characterized by precise motion control, adaptive feedback capabilities, and patient-interactive functions, are emerging as potential adjunctive tools within modern intensive care rehabilitation systems [75].

**Fig. 6.**
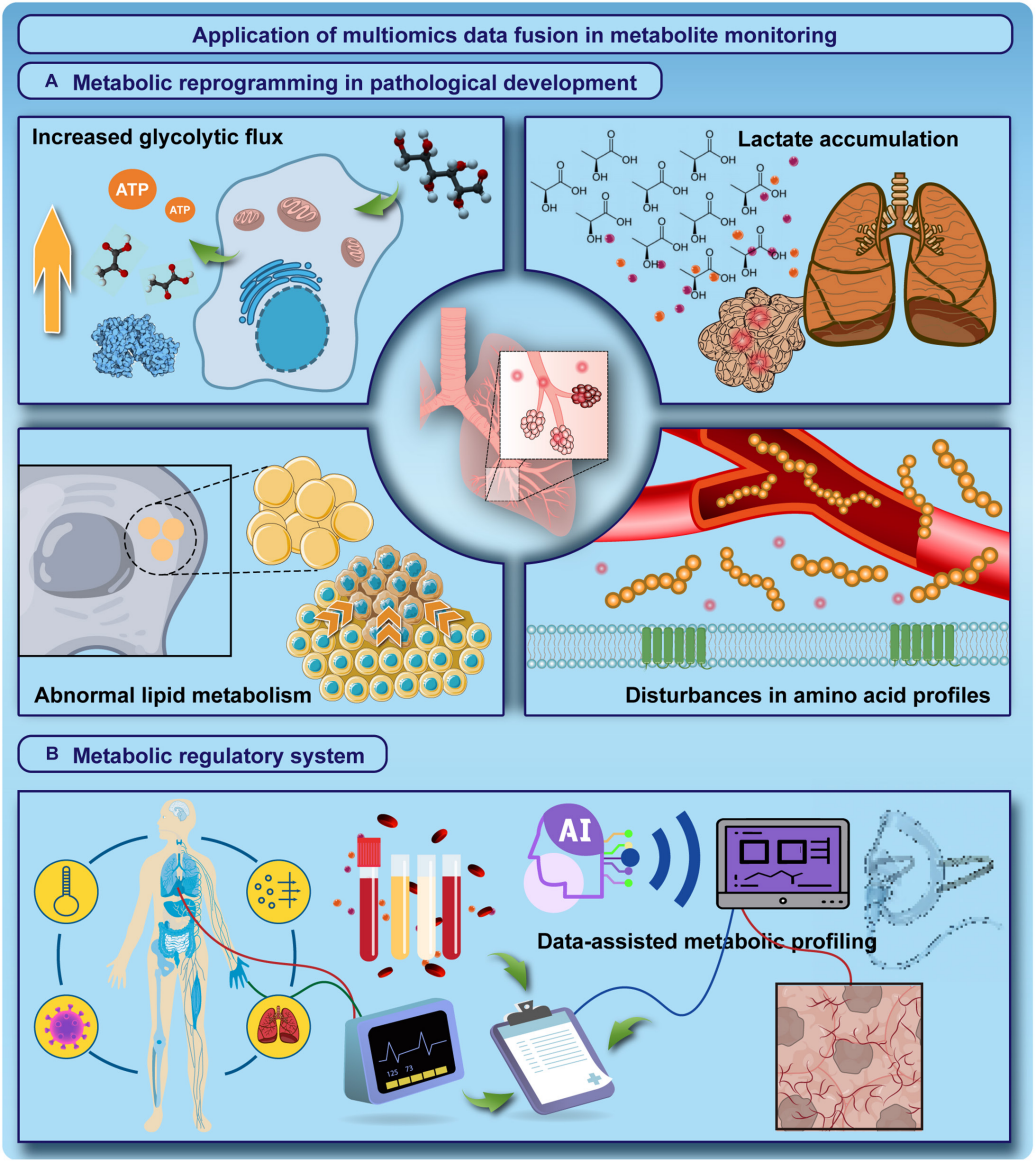
Application of multiomics data fusion in metabolite monitoring. (A) Metabolic reprogramming in pathological development: This module examines how metabolic changes contribute to the progression and recovery of severe pulmonary diseases. Increased glycolytic flux and lactate accumulation alter the tissue microenvironment, while disrupted lipid and amino acid profiles hinder cellular function. Together, this metabolic reprogramming modulates immune responses, inflammation resolution, and tissue repair. (B) Metabolic regulatory system: This section describes how multiomics data are used for personalized care. Data-assisted metabolic pattern analysis supports longitudinal monitoring by integrating blood metabolites, lung function, and exercise-related data. It helps optimize diagnosis, treatment, and rehabilitation.

Recent studies have demonstrated that metabolic dysregulation constitutes a core mechanism in the progression and recovery of pulmonary diseases. These metabolic alterations typically include increased glycolytic flux, lactate accumulation, lipid metabolism imbalance, and disturbances in amino acid profiles (Fig. 6) [76]. Such metabolic reprogramming not only reflects underlying pathophysiological processes but also modulates immune responses, inflammation resolution, and tissue regeneration. Metabolomics, as a major domain within systems biology, enables high-throughput and quantitative detection of small-molecule metabolites, providing critical insights into disease states, treatment responses, and potential therapeutic targets [77]. The integration of metabolomics with transcriptomic, proteomic, and clinical phenotype data allows for the construction of comprehensive multiomics maps that elucidate the molecular networks and functional dynamics of critically ill patients [78]. These multidimensional datasets provide a theoretical and data-informed basis for exploring how rehabilitation strategies assisted by robots may be informed and refined in future clinical applications. By monitoring biomarkers such as lactate, glutamine, fatty acids, branched-chain amino acids, and short-chain fatty acids across multiple biological fluids, including plasma, exhaled breath condensate, and bronchoalveolar lavage fluid, researchers can characterize metabolic trajectories associated with the acute, subacute, and convalescent phases of severe pulmonary disorders (Fig. 6). Measurements based on bronchoalveolar lavage fluid are mainly applicable in research contexts or selected clinical settings, and are not intended for routine bedside or real-time monitoring. Together, these dynamic metabolite profiles serve as indirect indicators of pulmonary functional recovery and provide mechanistic insight into immune activation, inflammatory status, and skeletal muscle metabolic turnover (Fig. 6).

Building upon these multiomics insights into metabolic dysregulation, an important challenge lies in translating such knowledge into actionable rehabilitation strategies. One important application of these insights is to guide the timing, intensity, and progression of robotic rehabilitation interventions. In the intensive care setting, real-time intervention is primarily guided by continuously or quasi-continuously available bedside physiological and surrogate metabolic signals, rather than direct incorporation of multiomics data. Multimodal sensor arrays integrated into robotic systems enable the acquisition of parameters such as heart rate, respiratory rate, electromyographic activity, and oxygen consumption, which form the basis for safety monitoring and fine-tuned adjustments during ongoing rehabilitation [79]. In contrast, multiomics data and data-driven predictive frameworks operate on longer time scales and are more suited to characterizing metabolic adaptability and recovery trajectories. Within this temporally stratified paradigm, real-time physiological signals support moment-to-moment control, while multiomics information provides a broader context for longitudinal optimization of rehabilitation strategies. This distinction enables clinically meaningful translation of metabolic information into practice. For example, if elevated lactate levels are observed without adequate cardiopulmonary compensation, rehabilitation strategies may be adjusted by clinicians or semiautomated systems to reduce physical load and extend recovery intervals.

**Fig. 7.**
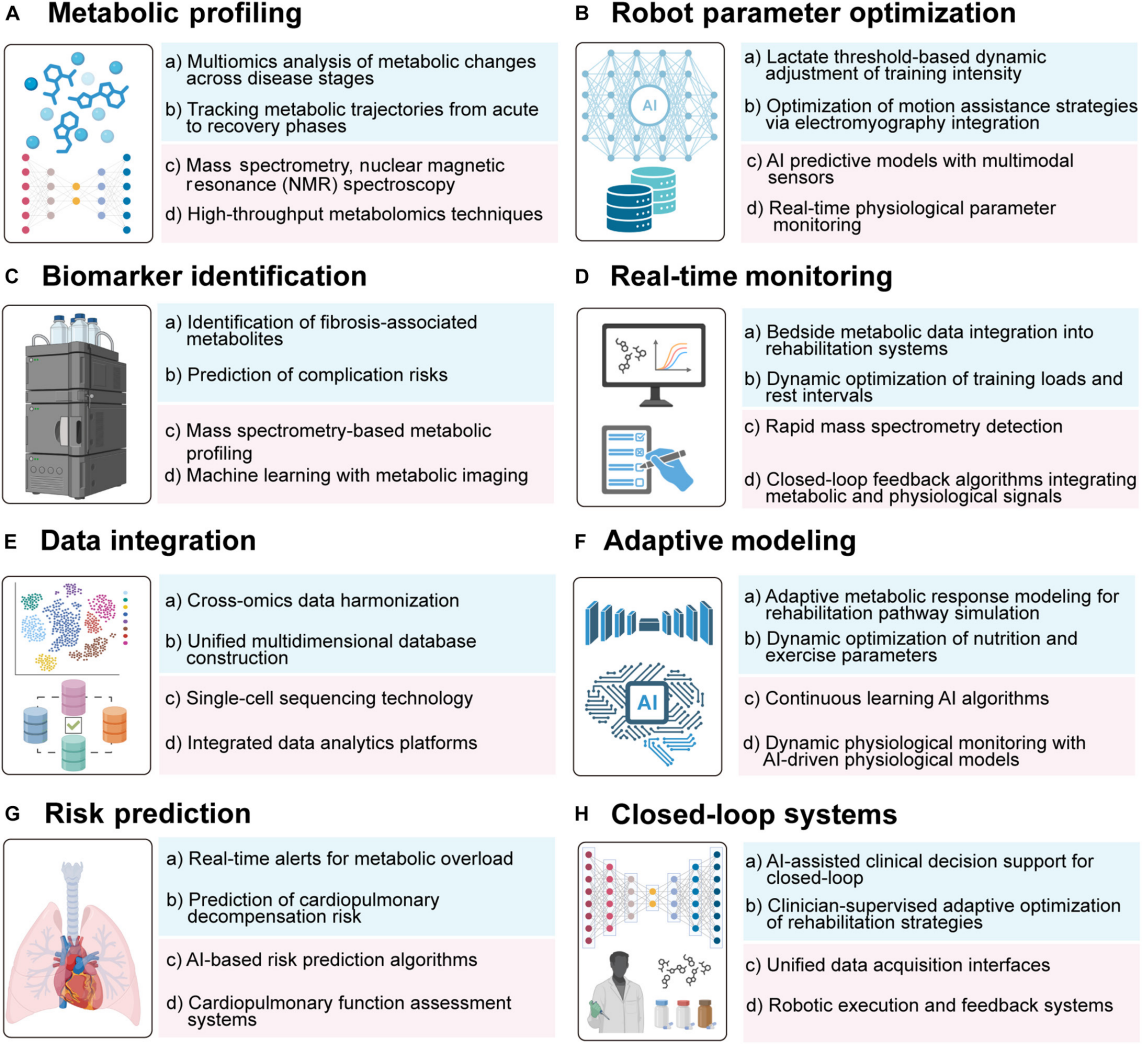
Integrated multiomics-guided framework for metabolic regulation and intelligent robotic rehabilitation. (A) Metabolic profiling: multiomics analysis of metabolic alterations and trajectories across disease stages. (B) Robot parameter optimization: AI-driven dynamic adjustment of training intensity and motion assistance strategies. (C) Biomarker identification: detection of fibrosis-associated metabolites and risk prediction via mass spectrometry and machine learning. (D) Real-time monitoring: continuous integration of bedside physiological and surrogate metabolic data to support feedback-informed adjustment. (E) Data integration: cross-omics data harmonization and construction of unified multidimensional databases. (F) Adaptive modeling: refinement of rehabilitation pathways through continuous learning algorithms and dynamic physiological monitoring. (G) Risk prediction: early warning systems for metabolic overload and cardiopulmonary decompensation. (H) Closed-loop systems: a synchronized execution framework linking data acquisition, modeling, and robotic actuation for individualized intervention.

Despite these advancements, the integration of heterogeneous multiomics and clinical data presents considerable technical challenges. The pathological mechanisms of pulmonary diseases exhibit strong spatial and temporal heterogeneity, rendering single-omics approaches insufficient for capturing the full complexity of disease progression [80]. Consequently, it is essential to establish unified, standardized, and continuously updated data integration platforms. These platforms must harmonize data from metabolomics, genomics, transcriptomics, physiological monitoring, and clinical evaluations (Fig. 7). Figure 7 provides an overview of the data flow involved in real-time monitoring, computational analysis, and robotic intervention, underscoring the conceptual importance of closed-loop regulatory strategies rather than fully implemented clinical systems. Such systems would be informed by metabolic profiles obtained through advanced techniques including mass spectrometry, nuclear magnetic resonance spectroscopy, and metabolic fingerprinting, which provide detailed biochemical assessments but are generally better suited to periodic or offline profiling than routine real-time bedside control [81].

In addition to molecular profiling, imaging modalities such as positron emission tomography and computed tomography contribute valuable spatial information by combining metabolic assessment with anatomical localization in lung lesions [82]. These images may support clinical assessment of areas of metabolic activity or fibrosis-related structural changes, thereby informing targeted adjustments in rehabilitation strategy. Integration of metabolic imaging data with omics-derived biomarkers and physiological signals may enhance the precision and adaptability of rehabilitation protocols. From a systems integration perspective, it is imperative that rehabilitation robots incorporate efficient data acquisition, fusion, and feedback execution mechanisms (Fig. 7). Interpretation of metabolomics data should be conducted in conjunction with parameters such as blood oxygen saturation and dynamic lung function indices, helping to ensure that robotic interventions are both timely and contextually appropriate (Fig. 7). When evidence of metabolic imbalance or energy supply–demand mismatch is detected, the system can recommend or support supervised adjustments to the training program to minimize physiological stress and support recovery-oriented management. Furthermore, AI algorithms enable the construction of predictive models that simulate individual patient responses based on multiomics and clinical input. These models could be iteratively updated using longitudinal and real-time clinical data, thereby supporting adaptive regulation and individualized intervention planning. In the future, rehabilitation robots are expected to function not merely as mechanical actuators but as supportive platforms that assist clinicians by providing metabolically informed references rather than replacing clinical decision-making (Fig. 7).

## Integration of Intelligent Rehabilitation Robots and Metabolite Feedback Systems

### Role of rehabilitation robots in supporting personalized metabolic management of critically ill pulmonary patients

Critically ill patients with pulmonary diseases frequently exhibit pronounced metabolic disturbances, including lactate accumulation, tissue hypoxia, and carbon dioxide retention. These metabolic abnormalities are largely driven by systemic inflammation, prolonged immobilization, and impaired respiratory mechanics, and they substantially compromise rehabilitation efficiency and clinical outcomes. In conventional rehabilitation settings, achieving precise and continuous metabolic monitoring and regulation remains challenging because of limited continuous metabolic assessment and insufficiently adaptable intervention strategies. In this context, rehabilitation robots may provide a supportive approach by facilitating personalized and adaptively adjustable rehabilitation strategies through integrated sensing, monitoring, and assistance technologies.

**Fig. 8.**
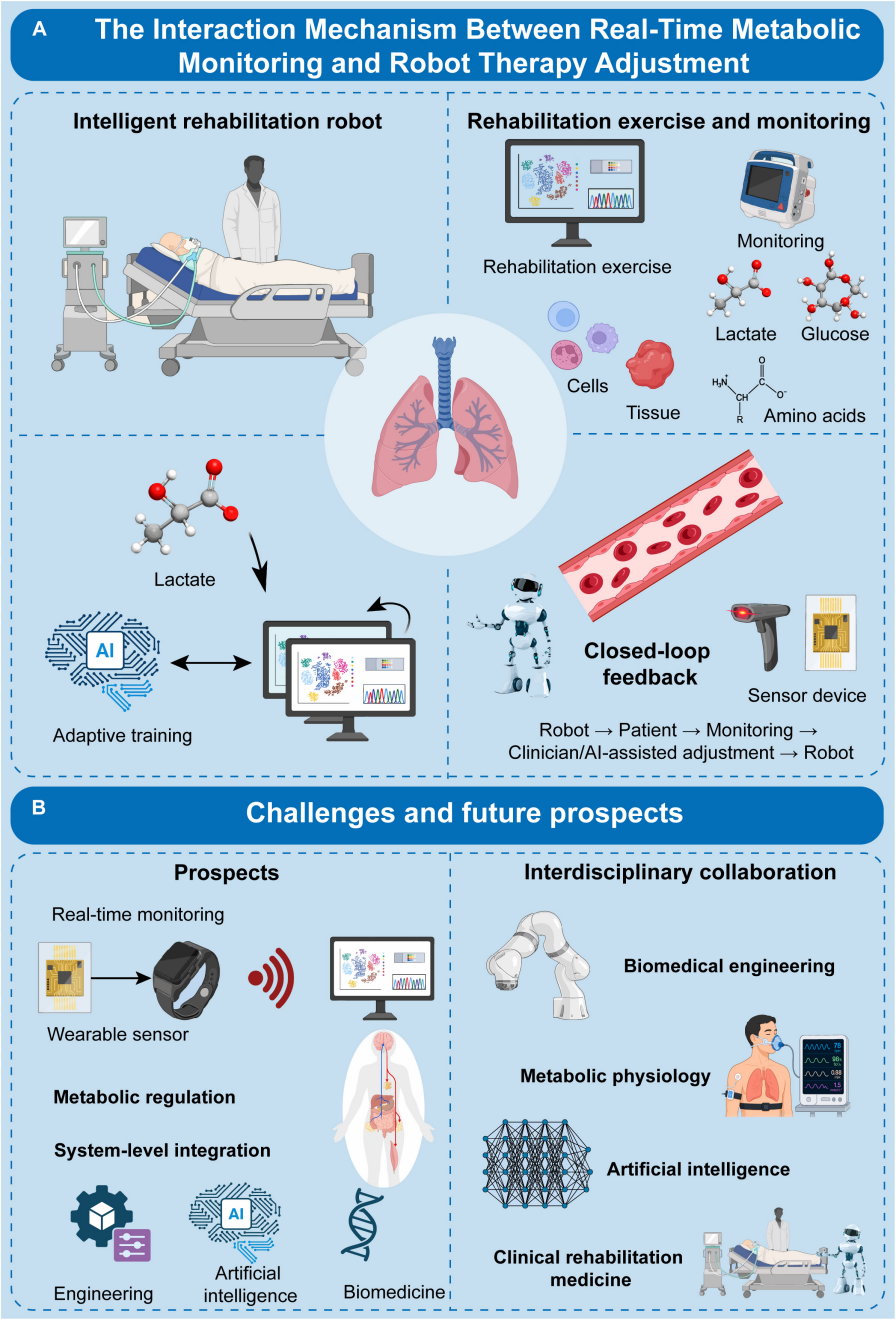
The integration of intelligent rehabilitation robots and metabolite feedback systems. (A) Interaction between real-time metabolic monitoring and robot-assisted therapy adjustment: This module shows the integration of rehabilitation robots with real-time metabolic monitoring. The system tracks metabolites, such as lactate, and adjusts therapy based on these data. It may support the adjustment of exercise intensity for personalized rehabilitation, with the aim of supporting metabolic regulation during recovery. (B) Challenges and future prospects: This section highlights challenges and future directions. Key areas include improving real-time monitoring and developing synchronized systems combining robotics, AI, and clinical expertise. Addressing these challenges may help improve rehabilitation effectiveness and expand its healthcare applications.

Rehabilitation robots can integrate multimodal physiological and surrogate metabolic indicators, including the respiratory exchange ratio, heart rate variability, blood lactate concentration, and muscle oxygen saturation. Collectively, these parameters provide information on metabolic load, energy utilization, and exercise tolerance. Such indicators can be dynamically acquired through portable metabolic measurement systems and bedside monitoring devices. When incorporated into clinical rehabilitation workflows, real-time data derived from these signals may support adjustments in exercise intensity, frequency, and modality under clinician supervision or through semiautomated robotic assistance. This data-informed approach may facilitate individualized rehabilitation planning while supporting procedural safety and precision (Fig. 8) [83]. For example, during robot-assisted gait or limb training, a rapid increase in lactate levels combined with unstable cardiopulmonary indicators may indicate excessive metabolic stress or insufficient oxygen delivery. In such cases, training intensity may be reduced and recovery intervals may be extended to prevent further metabolic imbalance. Conversely, stable metabolic parameters and preserved muscle oxygen utilization may support a gradual increase in training load with the aim of supporting metabolic adaptation and functional recovery. These bidirectional adjustments reflect a feedback-informed control process in which continuously acquired physiological and surrogate metabolic signals are translated into modifications of controllable robotic parameters, such as assistance level, resistance, and work–rest scheduling. In practice, such adjustments are performed under well-defined safety constraints, typically governed by rule-based control mechanisms and, in some cases, supported by predictive or data-driven approaches. Owing to these physiological and safety constraints, these systems are primarily designed to assist clinical decision-making rather than function as fully autonomous interventions. This is particularly important in the intensive care setting, where interpretability, auditability, and safety remain essential considerations.

**Fig. 9.**
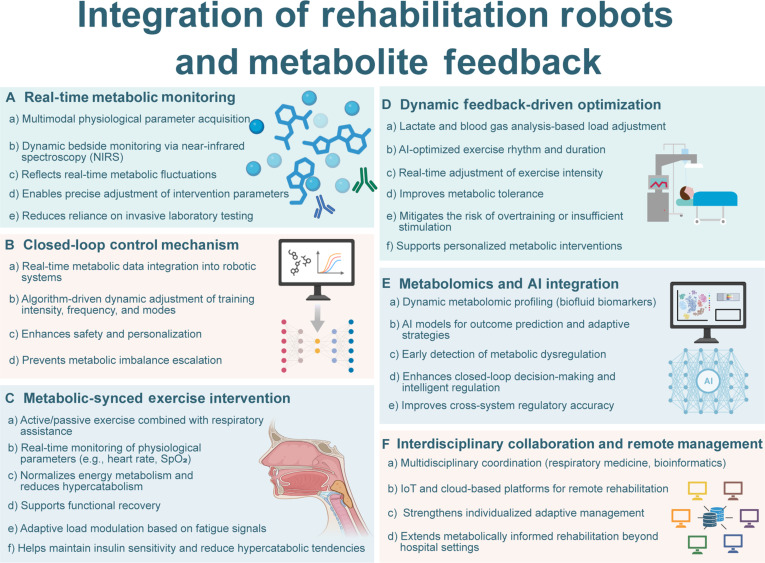
Integration of rehab robots and metabolite feedback. (A) Real-time metabolic monitoring: This module continuously monitors metabolic status using multimodal data, including near-infrared spectroscopy. It provides real-time insights, supporting precise adjustments to therapy, potentially reducing reliance on invasive testing, and personalizing care. (B) Closed-loop control mechanism: This system uses real-time physiological and surrogate metabolic data to adjust robotic rehabilitation parameters (intensity, frequency, and mode). It supports personalized therapy, improves safety, and helps reduce the risk of metabolic imbalance. (C) Metabolic-synced exercise intervention: This intervention combines exercise with respiratory support, monitoring heart rate and SpO_2_. It may help improve metabolic efficiency, alleviate hypercatabolic tendencies, and adjust intervention load according to fatigue signals to support recovery. (D) Dynamic feedback-driven optimization: Using real-time lactate and blood gas data, with AI-supported analysis, this approach adjusts exercise parameters. It supports rehabilitation optimization by potentially enhancing metabolic resilience and helping prevent overtraining. (E) Metabolomics and AI integration: Metabolomics and AI analyze biofluid biomarkers to predict outcomes and inform adaptive rehabilitation strategies. AI models may help detect early signs of metabolic dysregulation, supporting decision-making and therapy optimization. (F) Interdisciplinary collaboration and remote management: This system uses multidisciplinary coordination, supported by Internet of Things and cloud platforms, to enable remote rehabilitation. It extends metabolic monitoring and rehabilitation management beyond clinical settings, potentially improving rehabilitation continuity and patient outcomes.

With the advancement of rehabilitation medicine, metabolic assessment has progressively shifted from static laboratory-based evaluation toward more dynamic bedside monitoring approaches [84]. Given the rapid and often unpredictable metabolic fluctuations observed in critically ill pulmonary patients, timely interpretation of metabolic signals is essential. Rehabilitation robots equipped with data-assisted analytical frameworks can contribute to feedback-informed rehabilitation processes by linking real-time data acquisition with intervention guidance. This approach may help reduce reliance on subjective assessment and mitigate delays inherent in traditional manual adjustment, while supporting more data-informed and clinician-guided rehabilitation strategies. In addition to supporting intervention precision, rehabilitation robots may help address metabolic dysfunction associated with prolonged inactivity. By combining guided limb movements, respiratory assistance, and continuous physiological monitoring, these systems encourage skeletal muscle activation and energy utilization. Such interventions may contribute to improved metabolic efficiency and may help preserve insulin sensitivity while alleviating hypercatabolic tendencies related to immobility and inflammation (Fig. 9) [85]. During the training process, physiological parameters including heart rate, oxygen saturation, and respiratory rate can be evaluated alongside metabolic indicators such as lactate concentration and arterial blood gas profiles. This integrated information enables more informed adjustment of exercise load, rhythm, and duration.

Overall, the incorporation of metabolic feedback into robot-assisted rehabilitation supports clinician-guided optimization of training protocols. This approach helps reduce the risk of excessive physiological stress or insufficient stimulation during both early intervention and recovery phases. By tracking trends in metabolic indicators, rehabilitation robots may assist in identifying early signs of metabolic imbalance and alerting clinicians in a timely manner. This transition from purely mechanical exercise assistance toward metabolically informed rehabilitation support is particularly relevant in critically ill patients, in whom timely recognition of metabolic tolerance limits and transitions toward excessive anaerobic stress is critical for supporting the restoration of metabolic homeostasis and improving rehabilitation efficiency [86].

### Interdisciplinary collaboration and metabolically informed rehabilitation intervention

Traditional rehabilitation models are increasingly insufficient to address the complex and dynamic metabolic regulation requirements observed in critically ill patients with pulmonary diseases. The development of rehabilitation robot technologies has introduced more controllable and sustainable intervention modalities into clinical and translational rehabilitation settings [87]. At the same time, the integration of metabolomics and data-assisted analytical approaches is gradually shifting rehabilitation strategies from predominantly experience-dependent models toward more data-informed and individualized frameworks. This evolution reflects an emerging trend of interdisciplinary collaboration in critical care rehabilitation (Fig. 8).

Following the acute phase of critical illness, patients with severe pulmonary disorders commonly experience persistent metabolic dysregulation, skeletal muscle wasting, and reduced oxygen utilization efficiency. Through structured exercise guidance and real-time physiological feedback, rehabilitation robots can provide safe, controllable, and individualized rehabilitation support aimed at supporting oxygen delivery, promoting energy metabolic stability, and helping reduce secondary complications associated with prolonged bed rest. Compared with conventional passive rehabilitation approaches, robot-assisted rehabilitation offers advantages in exercise intensity modulation and training adherence, thereby providing a more precise platform for metabolically informed intervention [88].

The incorporation of metabolomics may further refine the targeting of rehabilitation interventions (Fig. 9). Dynamic monitoring of metabolite profiles in biological fluids may help identify metabolic disturbances that may indicate delayed recovery or increased risk of complications. When such information is interpreted together with robot-assisted rehabilitation data, intervention parameters such as exercise rhythm, intensity, and duration may be adjusted to better align with the patient’s metabolic tolerance. For instance, elevations in markers associated with protein catabolism may prompt conservative modification of exercise load to avoid exacerbating muscle metabolic stress, thereby contributing to a feedback-informed rather than fully autonomous regulatory process. Data-assisted analytical techniques can further support the rehabilitation process by integrating physiological parameters, metabolic characteristics, and exercise responses into structured interpretative frameworks [89]. These approaches may assist clinicians in assessing rehabilitation progression and anticipating potential responses to intervention, enabling timely modification of rehabilitation strategies. Within this collaborative framework, rehabilitation robots function not merely as execution devices but as supportive nodes for data collection and feedback. They contribute to coordinated multidisciplinary rehabilitation planning rather than replacing clinical judgment. Interdisciplinary collaboration remains fundamental to the effective implementation of metabolically informed rehabilitation systems [27]. Integration across respiratory medicine, critical care, rehabilitation medicine, and bioinformatics ensures that robot-assisted metabolic interventions are grounded in physiological rationale and clinical feasibility [90]. Through collaborative interpretation of metabolomic and physiological data, multidisciplinary teams can jointly tailor rehabilitation strategies, enhancing adaptability, responsiveness, and individualization of patient management.

As technological capabilities continue to evolve, remote rehabilitation and continuous management strategies are gradually being incorporated into the care of critically ill pulmonary patients [91]. Supported by Internet of Things infrastructure and cloud-based data architectures, rehabilitation robot platforms may facilitate real-time data exchange and remote clinical oversight. This approach extends metabolic monitoring and rehabilitation support beyond the inpatient setting and may help support metabolic stability during post-discharge recovery, potentially reducing readmission risk and improving long-term functional outcomes [92]. In summary, the application of rehabilitation robots in metabolically informed rehabilitation for critically ill pulmonary patients is transitioning from stand-alone technical assistance toward a more integrated supportive care platform. Their combination with metabolomics and data-assisted analytical methods reflects a gradual shift toward individualized and multidisciplinary rehabilitation strategies, offering a clinically grounded pathway to support recovery and improve patient quality of life.

## Challenges and Future Outlook

### Current challenges and difficulties

Current research on metabolically informed robotic rehabilitation in critically ill pulmonary patients remains at an early and exploratory stage, with limited direct clinical evidence available. To date, no randomized controlled trials or systematic reviews have specifically evaluated this integrated approach. Existing studies have largely focused on robotic rehabilitation and metabolic regulation as separate domains, rather than as a unified framework. This fragmentation in the evidence base contributes to several key technical and translational challenges that continue to constrain its clinical implementation. At the system level, a major limitation lies in the insufficient capacity for individualized intervention. The metabolic status of critically ill pulmonary patients is highly heterogeneous, whereas most existing rehabilitation robots rely on standardized training protocols and relatively fixed operational parameters. This mismatch limits the ability to dynamically adapt rehabilitation strategies in response to evolving metabolic conditions, thereby constraining the precision and effectiveness of robot-assisted metabolically informed rehabilitation [93]. At the sensing level, effective metabolic regulation depends on the timely acquisition of relevant metabolic indicators. However, current clinical practice relies predominantly on laboratory-based testing, which is associated with delayed feedback, complex workflows, and limited temporal resolution. These constraints reduce its applicability in bedside settings where metabolic fluctuations can occur rapidly. In addition, existing sensor technologies remain limited in sensitivity, long-term stability, and the ability to capture multiple parameters simultaneously. Continuous and minimally invasive metabolic monitoring systems that can be seamlessly integrated into rehabilitation robots have not yet been fully established. At the data processing level, even when high-frequency physiological and metabolic data are available, translating these signals into clinically actionable information remains a major challenge. Current analytical frameworks are often insufficient to extract meaningful patterns that can directly inform adaptive adjustment of rehabilitation parameters [94].

Although AI-based approaches offer potential advantages in data analysis and pattern recognition, their applicability and reliability in the rehabilitation of critically ill pulmonary patients remain limited [95]. On the one hand, the development of robust algorithmic models requires large-scale, well-annotated, and structurally standardized clinical datasets. In reality, data heterogeneity across institutions, inconsistencies in data quality, and constraints related to data privacy substantially limit data integration and sharing. On the other hand, many existing models focus primarily on macroscopic trend prediction and show limited responsiveness to subtle metabolic fluctuations. This limitation reduces their interpretability and restricts their practical value in clinical rehabilitation decision support [96]. From a system perspective, current rehabilitation robots continue to face challenges related to integration level and environmental adaptability, which hinder their widespread deployment in complex clinical environments [97]. In bedside settings for critically ill patients, factors such as device size, operational complexity, and response latency can substantially affect intervention safety and feasibility. In addition, many systems still rely heavily on manual parameter adjustment by healthcare professionals, rather than automated or semiautomated support, which increases workload and introduces potential variability in intervention quality [98]. At the control and modeling level, existing rehabilitation strategies are predominantly based on predefined, task-oriented frameworks that are insufficient to address the nonlinear and evolving nature of metabolic changes during recovery. In particular, the relationships between metabolic indicators and training load are rarely incorporated into control logic. The lack of integrated consideration of multiple physiological and surrogate metabolic parameters, including oxygen saturation, lactate concentration, and heart rate variability, further limits the development of feedback-informed intervention strategies. As a consequence, truly adaptive rehabilitation remains difficult to achieve in current systems. This limitation has highlighted the need to integrate multimodal physiological and surrogate metabolic signals into more interpretable feedback control frameworks, which are increasingly regarded as a key direction for improving system responsiveness and safety. Such approaches are particularly relevant in intensive care settings, where transparency, auditability, and clinical supervision remain essential.

Overall, the application of rehabilitation robots in metabolically informed rehabilitation for patients with severe pulmonary diseases remains at an early exploratory stage. Future progress requires coordinated advancement in 3 key areas. First, the development of metabolic monitoring technologies capable of providing multiparameter, real-time, and minimally disruptive feedback is essential. Second, intervention strategies must be guided by individualized metabolic response characteristics to improve adaptability and dynamic adjustment capacity. Third, system-level integration and intelligent coordination between perception, analysis, and execution need to be strengthened. Only through sustained multidisciplinary collaboration can the clinical potential of rehabilitation robots in metabolic management be more fully realized, thereby supporting safer, more precise, and effective rehabilitation for critically ill pulmonary patients.

### Workforce readiness and care continuum challenges

Beyond technical challenges, the real-world scalability and accountability of AI-enabled closed-loop rehabilitation systems depend critically on two often underdeveloped translational pathways: workforce readiness and continuity of care. Addressing these dimensions is essential for bridging the gap between technological innovation and routine clinical implementation. From the perspective of workforce development, the integration of AI into rehabilitation robotics introduces new competency requirements for clinicians, nurses, and trainees. These evolving demands highlight the need for a structured competency framework to ensure safe and effective system use. Core competencies include foundational AI literacy, recognition of algorithmic bias, understanding of model interpretability and uncertainty, and awareness of decision boundaries. Building on these competencies, clinical teams must also be prepared to manage system alerts, interpret human–machine interactions, comply with data privacy and regulatory requirements, and maintain standardized documentation and auditing practices. Such capabilities are particularly critical in intensive care settings, where misinterpretation of system outputs may compromise patient safety. To support skill acquisition and standardization, simulation-based and interprofessional training programs have been proposed. These programs may incorporate objective structured clinical examination-style assessments to evaluate operational proficiency, clinical interpretation, and safety response. From a system safety perspective, clear governance structures are required to ensure consistency and reliability across institutions. This includes defining intervention thresholds and escalation pathways, as well as systematically addressing potential failure modes such as data drift, sensor malfunction, algorithmic bias, and abnormal alerts. Accordingly, fallback protocols should be established to enable rapid transition to manual control or simplified rule-based strategies when system reliability is compromised. Within this context, AI-enabled rehabilitation systems are more appropriately implemented within a clinician-in-the-loop paradigm, emphasizing transparency, auditability, and accountability rather than full autonomy.

In parallel, the clinical value of AI-assisted rehabilitation robotics depends on its integration across the continuum of care, extending from the ICU to post-discharge recovery. During the acute phase in the ICU, AI-assisted systems may support risk stratification, assessment against rehabilitation initiation and discontinuation criteria, and safety gating, which should be aligned with key clinical parameters such as sedation level, delirium status, hemodynamic stability, and oxygenation. During the recovery phase, rehabilitation strategies should be coordinated with broader metabolic and supportive care domains, including nutritional support, glycemic control, sleep regulation, and analgesia, with training intensity adjusted according to the patient’s current metabolic and physiological tolerance. Beyond hospitalization, wearable devices and remote monitoring systems enable continuous tracking of physiological signals and patient-reported outcomes. These data support stratified follow-up, adherence management, and early identification of patients at increased risk of readmission. Such integration extends metabolically informed rehabilitation beyond the hospital setting and contributes to the development of continuous, patient-centered care pathways.

### Future development direction and technology outlook

Rehabilitation robot technology is gradually demonstrating its clinical relevance in the management of critically ill patients with pulmonary diseases, particularly in relation to metabolically informed rehabilitation strategies. With continued advances in interdisciplinary technologies, robot-assisted rehabilitation is evolving from a predominantly mechanical intervention approach toward a more integrated support framework that incorporates metabolic monitoring, data-assisted feedback, and adaptive rehabilitation planning [99]. This evolution reflects a broader shift in rehabilitation concepts and highlights the increasing importance of personalization informed by metabolic status in critical care rehabilitation.

At present, rehabilitation robots that integrate physiological monitoring systems are capable of capturing dynamic changes in patient status and providing continuous physiological data to support clinical assessment [100]. This capability enables rehabilitation strategies to move beyond static evaluation toward more responsive and individualized adjustment. In patients with pulmonary diseases complicated by respiratory dysfunction and systemic metabolic imbalance, such data-informed support may assist clinicians in tailoring intervention intensity and pacing according to indicators related to fatigue, oxygenation, and energy utilization, thereby improving rehabilitation safety and feasibility rather than implying fully autonomous optimization [14].

Advances in robotic technology are also influencing perioperative and postoperative rehabilitation pathways for patients with severe pulmonary diseases. Although surgical robots are primarily applied in operative settings, their development has contributed to a broader trend toward minimally invasive and precision-oriented treatment strategies [101]. These changes place increased emphasis on early postoperative rehabilitation. In this context, rehabilitation robots may support structured functional training and metabolic management during early recovery phases, with the aim of reducing complications and supporting quality of life rather than replacing conventional clinical management.

Future development may focus on enhancing the intelligent capabilities of rehabilitation robot systems through the incorporation of data-assisted analytical methods [102]. Rather than executing fixed intervention programs, such systems may support the adaptive adjustment of rehabilitation plans based on integrated interpretation of metabolic, physiological, and functional data. Multimodal sensing and feedback mechanisms may further improve system responsiveness and safety in human–machine interaction. However, these developments should be viewed as incremental advancements rather than fully realized autonomous systems.

The realization of these goals depends fundamentally on multidisciplinary collaboration. The convergence of biomedical engineering, metabolic physiology, AI research, and clinical rehabilitation medicine is expected to drive gradual innovation in rehabilitation robot systems [103]. Establishing standardized and quantifiable rehabilitation assessment metrics will be essential for evaluating intervention effectiveness and facilitating clinical translation. In parallel, advances in metabolomics may provide deeper insight into metabolic dysregulation patterns in critically ill pulmonary patients, thereby offering more refined reference information for rehabilitation planning [104].

Despite ongoing challenges related to cost, system integration, and individualized adaptation, the role of rehabilitation robots in the critical care management of pulmonary diseases is expected to expand as technologies mature and clinical experience accumulates. Advances in wearable sensing technologies and data transmission infrastructure may further enable these systems to support remote monitoring and post-discharge rehabilitation management. Such developments could extend metabolically informed rehabilitation beyond hospital settings and contribute to the establishment of continuous, patient-centered rehabilitation pathways [105]. Within this evolving landscape, rehabilitation robotics in metabolic management should be viewed not as a fully established therapeutic paradigm, but as an emerging framework that integrates metabolic awareness, clinician-guided decision support, and multidisciplinary collaboration. This perspective reflects a gradual transition from auxiliary technical support toward more integrated and context-aware rehabilitation strategies. Further methodological refinement and clinical validation will be essential to determine the long-term impact of this approach on rehabilitation quality and patient outcomes. Future research should prioritize the development of structured validation frameworks, including assessments of feasibility and safety, such as adverse events and hemodynamic tolerance, as well as evaluations of system usability and nursing workload. In addition, randomized controlled trials comparing AI-assisted robotic rehabilitation with standard robotic rehabilitation or usual care will be necessary, alongside clearly defined clinical endpoints and robust data governance strategies.

## Conclusion

Metabolic reprogramming has been increasingly recognized as a central biological process underlying recovery from severe pulmonary dysfunction. This process is closely linked to exercise-induced metabolic adaptations, which contribute to the restoration of cellular energy homeostasis and the promotion of protein synthesis. Alterations in metabolic indicators, including lactate, amino acids, and short-chain fatty acids, reflect these adaptive responses and are involved in the regulation of immune–metabolic interactions that support tissue repair and pulmonary recovery. These interconnected metabolic and immune processes highlight a physiologically grounded pathway through which rehabilitation interventions may exert therapeutic effects. Within this context, rehabilitation robots can be understood as a means of delivering controlled and quantifiable exercise stimuli that facilitate such metabolic adaptations. When combined with metabolic monitoring platforms, these systems enable dynamic adjustment of training intensity and frequency based on individual physiological responses. This adaptive regulation may help maintain energy balance and improve the efficiency of recovery.

AI-enabled rehabilitation robotic systems have introduced a new technical framework for metabolically oriented management in critically ill patients with pulmonary diseases. By integrating intelligent sensing, adaptive control, and continuous feedback, these systems enable closer coupling between physiological monitoring and individualized therapeutic regulation than conventional rehabilitation approaches. By continuously collecting and analyzing cardiopulmonary and metabolic parameters, rehabilitation robots can support the delivery of sustained and quantifiable training stimuli that promote muscle activation, a process that has been associated with improvements in mitochondrial function and substrate utilization, including glucose and lipid metabolism. Collectively, these functions may contribute to the maintenance of systemic metabolic homeostasis, modulation of inflammation-related metabolic stress, and functional recovery of respiratory and skeletal muscle systems. Looking ahead, rehabilitation robots are expected to gradually extend beyond mechanical assistance toward more integrated systems that combine metabolic monitoring, multimodal sensing, and data-informed decision support. Their capacity for individualized feedback, continuous data acquisition, and data-driven adaptation establishes a foundation for precision rehabilitation within critical care. The convergence of robotics, bioinformatics, systems physiology, and clinical medicine will further advance this field, leading to an interconnected, patient-centered framework for metabolic rehabilitation. This evolving paradigm highlights the potential role of interdisciplinary collaboration in advancing metabolic-oriented rehabilitation strategies for pulmonary critical illness and provides a conceptual reference for future research and clinical exploration.
